# Preoperative Corticosteroid Injection and Postoperative Infection Following De Quervain’s Release: A National Database Analysis

**DOI:** 10.1177/15589447261437830

**Published:** 2026-05-11

**Authors:** Mikayla P. Borusiewicz, Thompson Zhuang, David R. Steinberg

**Affiliations:** 1 Hospital of the University of Pennsylvania, Philadelphia, USA

**Keywords:** corticosteroid, De Quervain’s tenosynovitis, infection, diagnosis, injection, glucocorticoid, wrist, anatomy, outcomes, research & health outcomes

## Abstract

**Background:**

De Quervain’s tenosynovitis is initially treated with analgesics, immobilization, and corticosteroid injections. Patients with symptoms refractory to corticosteroid injection may require surgical release. No prior studies have identified whether injection of corticosteroid for De Quervain’s tenosynovitis leads to adverse postoperative outcomes. This study aimed to determine whether corticosteroid injections administered within 3 months prior to De Quervain’s release increase the risk of postoperative surgical site infection.

**Methods:**

We used a national administrative database to identify adult patients undergoing De Quervain’s release and divided patients into two cohorts based on whether they received a corticosteroid injection for De Quervain’s tenosynovitis within 90 days prior to the surgery. After propensity score matching for age, sex, US region, insurance plan, Elixhauser Comorbidity Index, and history of tobacco use, rates of surgical site infection, reoperation, and wound dehiscence were assessed.

**Results:**

Patients who received an injection within the 90-day period prior to surgical release did not experience a higher incidence of surgical site infections, return to the operating room for infection, or wound dehiscence. An additional sensitivity analysis assessing the risk of injection within 60 days before surgery showed no increased risk of postoperative surgical site infection.

**Conclusions:**

Injection of corticosteroids within 60 or 90 days prior to surgical release for De Quervain’s tenosynovitis was not associated with increased risk of postoperative surgical site infection or return to the operating room to treat infection.

## Introduction

De Quervain’s tenosynovitis describes the painful tendinopathy caused by inflammation around and thickening of the first dorsal extensor compartment over the radial styloid. This fibrous tunnel houses the abductor pollicis longus and extensor pollicis brevis tendons as they course to their respective insertions on the base of the thumb metacarpal and proximal phalanx. The hypothesized pathophysiologic basis implicates friction produced by repetitive thumb abduction combined with ulnar deviation of the wrist. This may perpetuate a cycle of inflammation and narrowing of the fibro-osseous tunnel, leading to characteristic pain over the first dorsal compartment. Initial management is nonsurgical, including rest, activity modification, nonsteroidal anti-inflammatory medications, and splinting.^1,2^ For those with refractory symptoms, corticosteroid injection into the painful tendon sheath and subsheaths may provide relief.^3,4^ Prior studies report symptom resolution in 50% to 83% of patients after a single injection, with additional improvement in 66% to 70% of those receiving subsequent injections.^1,2,5-8^ Nevertheless, for the subset of patients whose symptoms fail to resolve following either single or multiple corticosteroid injections, surgical release of the first dorsal compartment including any present subsheaths remains definitive management.^2,3^

While corticosteroid injections are widely used for various orthopedic conditions for their anti-inflammatory effects, potential risks should be considered. Several studies have evaluated the relationship between perioperative injections and surgical site infection for multiple upper-extremity procedures, namely trigger finger release, thumb carpometacarpal arthroplasty, and carpal tunnel release.^5,9-12^ While one study demonstrated no temporal relationship between timing of injection and surgical site infection after open trigger finger release,^11^ other studies have reported an increased risk of infection when injections were administered at variable time points, ranging from 30 to 90 days prior to surgery.^12,13^ Similarly, there is no consensus on a safe window between corticosteroid injection and carpal tunnel release to minimize risk of infection, with recommendations ranging from 30 to 90 days^14,15^; another study found no temporal relationship between injection and surgical site infection after carpal tunnel release.^16^

For De Quervain’s tenosynovitis specifically, no consensus exists within the available literature regarding a safe interval between preoperative corticosteroid injection into the first dorsal extensor compartment and surgical release. No prior studies have evaluated the effects of timing of preoperative steroid injection on postoperative outcomes in patients with De Quervain’s tenosynovitis. We hypothesized that wrist injections may carry a lower postoperative infection risk than digital injections, as the larger cross-sectional area and longer tendon sheaths at the wrist may allow for greater diffusion of the steroid’s effect. The purpose of this study was to identify whether receipt of a corticosteroid injection prior to De Quervain’s release increases the risk of postoperative surgical site infection.

## Materials and Methods

### Data Source

**Figure 1.**
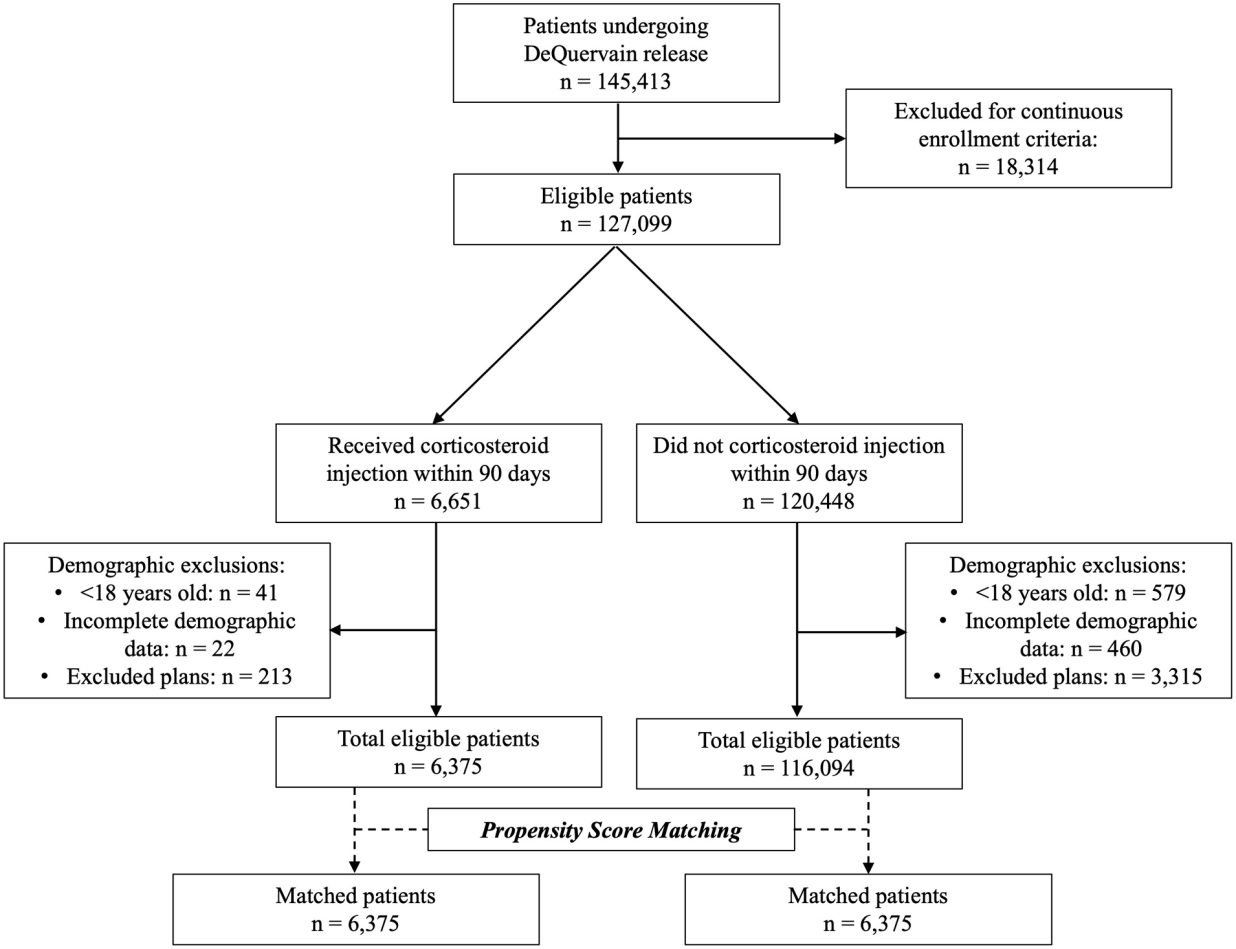
Flow diagram of cohort creation.

We utilized data from a national administrative claims database (PearlDiver Technologies, Inc.), which contains diagnostic, procedural, and demographic records from over 170 million patients. Demographic data included patient age, sex, US region, and insurance type. Diagnostic data such as comorbidities are included via International Classification of Diseases, Ninth and Tenth Revision (ICD-9/10) diagnostic codes, and surgical procedures are designated using Current Procedural Terminology (CPT) codes. We identified all patients undergoing De Quervain’s release using its CPT code (CPT-25000). If patients had more than one De Quervain’s release, we only included the first instance. We required that patients be actively enrolled in the database continuously for at least 1 year prior to and 90 days after De Quervain’s release to accurately capture comorbidity and outcome data, respectively. Patients were divided into cohorts depending on whether they received a corticosteroid injection for De Quervain’s tenosynovitis within 90 days preoperatively. The timing of injection was determined by the most recent injection relative to surgery. In a sensitivity analysis, we defined the exposure as receiving a corticosteroid injection for De Quervain’s tenosynovitis within 60 days preoperatively. We were unable to consider the number of injections. Corticosteroid injections were defined by the co-occurrence of a CPT code for a tendon sheath injection, an administration code for an included corticosteroid, and a diagnostic code for De Quervain’s tenosynovitis (Supplemental Materials, Table S1). Since the CPT code for tendon sheath injection might also be used for trigger finger injections, we excluded all injections occurring on the same day with an associated diagnostic code for trigger finger (Supplemental Materials, Table S1). Included corticosteroids were triamcinolone acetonide, triamcinolone diacetate, triamcinolone hexacetonide, methylprednisolone acetate, betamethasone acetate, betamethasone sodium phosphate, dexamethasone sodium phosphate, and dexamethasone acetate. We only included patients who were at least 18 years of age and with private, Medicare Advantage, or Medicaid-managed care insurance plans. Fee-for-service Medicare patients are not represented in this database. We required that patients have complete demographic data for inclusion. This is similar to prior published methodology.^17^ A flow diagram of cohort creation is shown in Figure 1.

### Variables

The primary explanatory variable was receiving a corticosteroid injection for De Quervain’s tenosynovitis within 90 days prior to surgical release. Patient age, sex, US region, primary insurance plan, and Elixhauser Comorbidity Index were reported as covariates. The primary outcome variable was the incidence of surgical site infection within 90 days after surgery. We defined this in two ways. First, we used ICD diagnostic codes corresponding to surgical site infection, which included codes explicitly referring to surgical site infection as well as superficial (eg, cellulitis) and deep (eg, osteomyelitis) infections. Then, we also used procedural codes corresponding to common procedures used for the treatment of surgical site infection (ie, irrigation and debridement) to identify return to the operating room for infection. All codes used are provided in Supplemental Materials, Table S1.

### Statistical Analysis

Patients with preoperative corticosteroid injections for De Quervain’s tenosynovitis and those without were propensity score matched using a logistic regression model to account for the following covariates, which were selected *a priori*: age, sex, US region, insurance plan, Elixhauser Comorbidity Index, and history of tobacco use. History of tobacco use was defined using ICD-9/10 codes. The Elixhauser Comorbidity Index is a validated measure of comorbidity burden that is commonly used in administrative database research. Notably, components of the Elixhauser Comorbidity Index include a prior history of cancer, human immunodeficiency virus (HIV) infection, rheumatoid arthritis, systemic lupus erythematosus, or malnutrition, which enter the matching algorithm. Matching was performed in a 1:1 ratio with a caliper width of 0.20. The caliper width denotes the maximum acceptable propensity score difference between pairs of matched participants. A caliper width of 0.2 has been considered optimal for maximizing the number of permissible matches while improving the closeness of match.^18^ Categorical variables were compared using chi-square tests. Differences in the Elixhauser Comorbidity Index were compared using the Mann-Whitney test. Differences in these demographic characteristics were compared prior to and after propensity score matching to evaluate success of match. Statistical significance was defined as *P* < .05.

Based on an overall surgical site infection incidence of ~1% within 90 days after soft-tissue hand surgery, we calculated that at least 2456 patients are needed in each cohort to detect at least a 1-percentage-point difference with 80% power (alpha = 0.05).^17^

## Results

**Figure 2.**
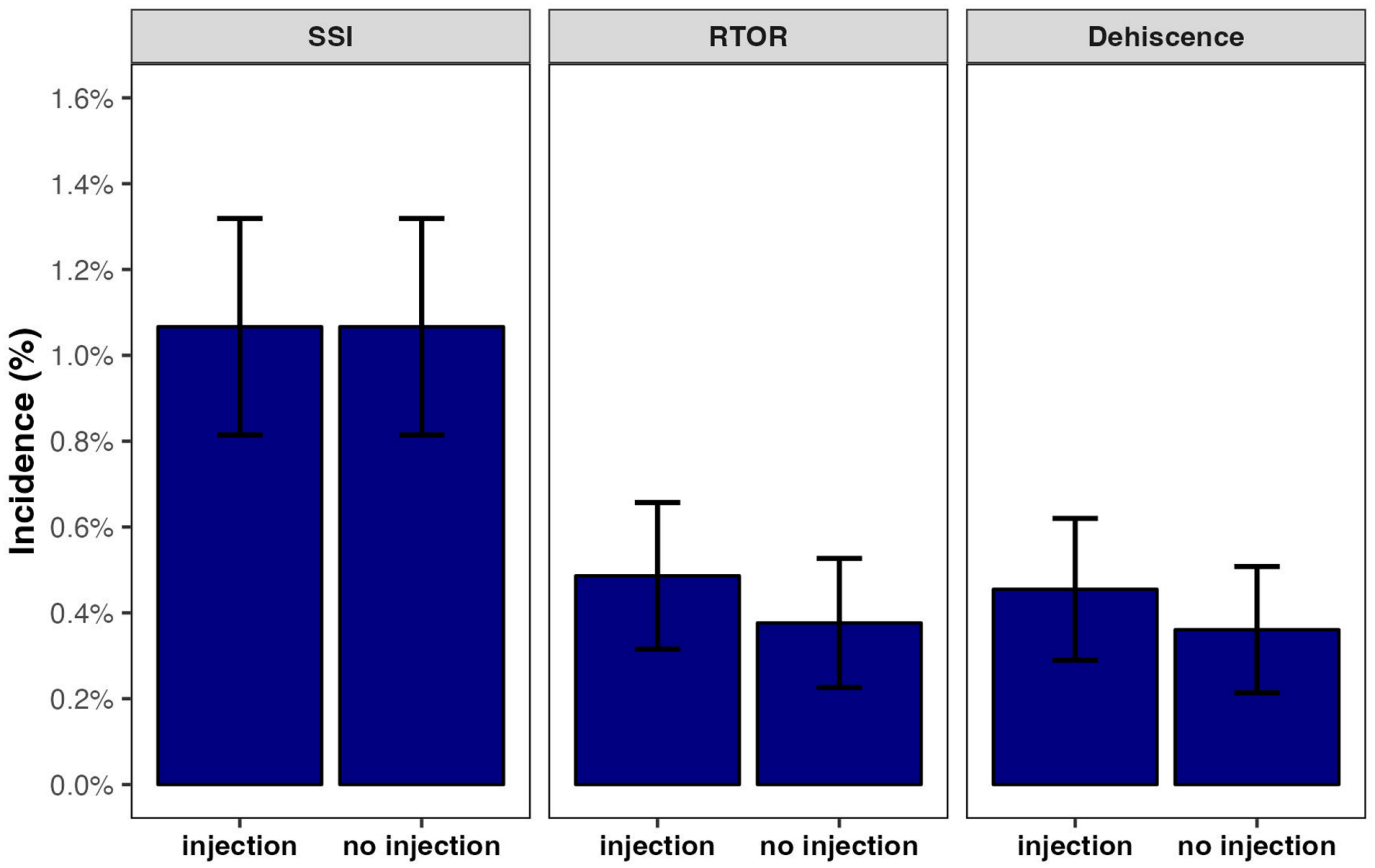
Incidence of complications after De Quervain’s release by cohort. *Note.* SSI = surgical site infection; RTOR = return to the operating room (for infection).

Of 122 469 eligible patients undergoing De Quervain’s release, 6375 (5.2%) received a corticosteroid injection for De Quervain’s tenosynovitis within 90 days before surgery. Patients who received an injection and those who did not differed with respect to age and geographical distributions. After propensity score matching, there were no residual differences in demographic factors between the cohorts (Table 1). Compared to patients who did not receive a corticosteroid injection within 90 days before surgery, patients who received an injection within 90 days before surgery did not experience a higher incidence of surgical site infections, return to the operating room for infection, or wound dehiscence (Table 2, Figure 2).

**Table 1.** Cohort Demographics Before and After Propensity Score Matching.

Variable	Before propensity score matching			After propensity score matching		
	Cohort		*P* value	Cohort		*P* value
	Injection (n = 6375)	No injection (n = 116 094)		Injection (n = 6375)	No injection (n = 6375)	
Age group, n (%)			<.001			.89
<45 years	1791 (28.1)	25 066 (21.6)		1791 (28.1)	1829 (28.7)	
45-54 years	1464 (23.0)	26 667 (23.0)		1464 (23.0)	1464 (23.0)	
55-64 years	1751 (27.5)	34 979 (30.1)		1751 (27.5)	1734 (27.2)	
≥65 years	1369 (21.5)	29 382 (25.3)		1369 (21.5)	1348 (21.1)	
Sex, n (%)			.15			.75
Female	5216 (81.8)	95 806 (82.5)		5216 (81.8)	5231 (82.1)	
Male	1159 (18.2)	20 288 (17.5)		1159 (18.2)	1144 (17.9)	
Region, n (%)			<.001			.99
Midwest	1608 (25.2)	31 212 (26.9)		1608 (25.2)	1612 (25.3)	
Northeast	1523 (23.9)	25 006 (21.5)		1523 (23.9)	1519 (23.8)	
South	2365 (37.1)	45 903 (39.5)		2365 (37.1)	2353 (36.9)	
West	879 (13.8)	13 973 (12.0)		879 (13.8)	891 (14.0)	
Insurance plan, n (%)			.24			.17
Private	4955 (77.7)	91 053 (78.4)		4955 (77.7)	5042 (79.1)	
Medicare	979 (15.4)	17 583 (15.1)		979 (15.4)	919 (14.4)	
Medicaid	441 (6.9)	7458 (6.4)		441 (6.9)	414 (6.5)	
Elixhauser Comorbidity Index, mean (*SD*)	3.84 (3.37)	3.85 (3.29)	.22	3.84 (3.37)	3.69 (3.16)	.16

*Note.* Cohorts were propensity score matched for age, sex, region, insurance plan, Elixhauser Comorbidity Index, and history of tobacco use. *SD* = standard deviation.

**Table 2.** Postoperative Outcomes for 90-Day Cohorts.

Outcome	Cohort		*P* value*
	Injection within 90 days	No injection within 90 days	
Surgical site infection, n (%)	41 (1.0)	45 (1.1)	.75
Reoperation for infection, n (%)	20 (0.5)	13 (0.3)	.30

* Signifies comparison between patients who did and did not receive corticosteroid injection within 90 days.

In the sensitivity analysis, 4164 patients (3.4%) received a corticosteroid injection for De Quervain’s tenosynovitis within 60 days before surgery (Supplemental Materials, Table S2). After propensity score matching, there were no residual differences in demographic factors between the cohorts. Compared to patients who did not receive a corticosteroid injection within 60 days before surgery, patients who received an injection within 60 days before surgery did not experience a higher incidence of surgical site infections or return to the operating room for infection. There was a higher incidence of wound dehiscence in patients who received an injection within 60 days before surgery. Wound dehiscence was defined using ICD-9-CM and ICD-10-CM diagnosis codes characterizing postoperative wound separation and included dehiscence at all levels of depth, as well as depths unspecified (Supplemental Materials, Table S3). Because this is a claims-based analysis, specific interventions for wound dehiscence could not be directly ascertained or individually analyzed.

## Discussion

In this study of a propensity-matched cohort from a large, national administrative database, receipt of a corticosteroid injection for De Quervain’s tenosynovitis within 2 or 3 months of first dorsal compartment release was not associated with an increased risk of superficial or deep surgical site infection or reoperation for infection compared to patients who did not receive a corticosteroid injection within that interval. The incidence of surgical site infection was nearly identical between the cohorts at approximately 1%, suggesting that corticosteroid injections may be administered within 60 or 90 days of surgery for De Quervain’s tenosynovitis without an increased risk of infection. The relatively higher incidence of wound dehiscence in patients who received an injection within 60 days before surgery warrants further study. The clinical significance of this finding is unknown, as the absolute risk of wound dehiscence remained low.

The relationship between perioperative corticosteroid injections and postoperative surgical site infections has been explored by several prior studies for manifold orthopedic conditions.^10-16^ Corticosteroids have well-established immunosuppressive properties, ushering concern for postoperative complications related to healing and pathogen clearance. These agents suppress inflammatory cytokines (eg, IL-1, IL-6, IL-12, and tumor necrosis factor) and inhibit macrophage apoptosis, neutrophil superoxide production, and the activity of metalloproteases.^19,20^ Injected corticosteroids downregulate local immune responses, and prior studies have demonstrated that these effects may be cumulative and systemic. The consequence of these effects is suppression of cell-mediated and humoral helper responses to local pathogens, both of which otherwise help to combat infectious processes.^20-22^

Multiple studies examining corticosteroid injections for upper-extremity conditions have demonstrated that decreased time between injection and surgery was a risk factor for postoperative infections.^14,15^ In a retrospective review of 999 digits undergoing trigger finger release, the authors identified steroid injection and fewer days between injection and surgery as risk factors for postoperative surgical site infection. Their results identified a linear relationship between time from injection to surgery and subsequent rates of infection and supported an 80-day preoperative period as the threshold outside of which the risk of postoperative infection was decreased.^13^ Another study demonstrated increased deep surgical site infections following trigger finger release with preoperative corticosteroid injection within 90 days.^5^ In contrast, a recent study of corticosteroid injections before carpal tunnel release found no increased risk of postoperative deep infection.^16^ Therefore, some clinical equipoise exists as to the risk of postoperative infection with preoperative corticosteroid injections in hand surgery.

Effective use of corticosteroid injections for nonsurgical treatment of De Quervain’s tenosynovitis has been well documented.^1,2^ In a retrospective study, 73.4% of affected extremities improved within 2 injections.^3^ A pooled quantitative review of 35 articles reported an 83% cure rate with injection alone, which was superior to injection combined with splinting, which yielded only a 61% success rate.^23^ A 2015 systematic review evaluated 5 studies and found that patients receiving injections for the treatment of De Quervain’s tenosynovitis had less pain and fewer limitations at their first follow-up after injection and higher rates of complete symptom resolution during the follow-up period.^1^ To date, no study has identified whether there is an increased risk of postoperative surgical site infection for patients receiving preoperative injections for De Quervain’s tenosynovitis. A recent study demonstrated that distant-site injections into large or intermediate joints did not increase 90-day risk of surgical site infection across multiple upper-extremity procedures, including De Quervain’s release, trigger finger release, and carpal tunnel release.^17^ However, that study evaluated intra-articular injections as opposed to injections into the first dorsal compartment. For patients who remain symptomatic after injection of corticosteroids for De Quervain’s tenosynovitis, our results offer evidence derived from a large claims data–based cohort that surgical release within 2 or 3 months of local corticosteroid injection does not pose an increased risk of postoperative infection.

In this study, only 5.2% of patients received a corticosteroid injection within the 90-day period prior to surgery. Several factors may explain this relatively low rate despite the well-documented efficacy of corticosteroid injections for De Quervain’s tenosynovitis. First, our cohort exclusively captures patients who progressed to surgical release and may therefore represent a selected population with refractory or severe symptoms. The study design excludes those whose symptoms resolved after any number of corticosteroid injections. Patients may have undergone conservative management or injection prior to referral to a subspecialty surgeon, further defining the surgical cohort for those with persistent or severe symptoms. Second, any patient who had undergone an ineffective injection more than 90 days prior to surgery or received multiple remote injections would have been classified within the noninjected group. Accordingly, the observed 5.2% preoperative injection rate reflects recent injections rather than lifetime injection exposure. Finally, injection rates in claims-based analyses may be influenced by the individual practice and coding patterns across providers and may not reflect the true prevalence of corticosteroid injections in the observed population.

Several limitations are innate to our study design and its reliance on claims data. The data are limited in granularity and depend on the accuracy of the billing process, although there exists an incentive for proper coding to ensure appropriate reimbursement. Less severe or subclinical infections may not have been captured by the clinical documentation and thus may have been underrepresented by the coded diagnoses. In addition, the limited data inhibits a detailed assessment of comorbidities that may influence risk of surgical site infection within our cohort. For example, limited sample sizes precluded a subgroup analysis of patients with diabetes mellitus, which is known to independently increase the risk of surgical site infection.^24,25^ Nevertheless, diabetes mellitus is included in the Elixhauser Comorbidity Index and was accounted for in the matching algorithm used. Future prospective studies might explore the association between preoperative injections and surgical site infections in this patient population.

Because serial injections could not be assessed, their cumulative effect on infection risk remains unclear. However, had an effect of serial injections and local immunosuppression been clinically meaningful, we would have observed an increased risk of postoperative infection in the treated subgroup. Furthermore, a 2020 retrospective review of 999 digits undergoing trigger finger release did not identify any relationship between multiple injections and postoperative infection, which may be extrapolated to patients with De Quervain’s tenosynovitis.^13^ Finally, our results could not account for individual surgeons’ practices or differences in corticosteroid type, dosage, preparation, and injection technique. Perioperative antibiotic prophylaxis and timing for dressing removal, suture removal, and return to work could have been heterogeneous. Notably, prior studies have shown that antibiotic prophylaxis is not needed for soft-tissue hand surgeries.^26^

Future studies of surgical site infection rates in patients undergoing corticosteroid injection for De Quervain’s tenosynovitis might examine shorter intervals within the postoperative period. Defining the minimum safe interval between injection and surgery as it pertains to infectious risk may facilitate more rapid relief for patients whose symptoms are refractory to corticosteroid injection.

## Conclusion

In conclusion, this large database study demonstrates that receiving a preoperative corticosteroid injection for De Quervain’s tenosynovitis within 60 or 90 days of surgical release was not associated with increased rates of postoperative surgical site infection or return to the operating room to treat infection.

## Supplemental Material

sj-docx-1-han-10.1177_15589447261437830 – Supplemental material for Preoperative Corticosteroid Injection and Postoperative Infection Following De Quervain’s Release: A National Database Analysis Supplemental material, sj-docx-1-han-10.1177_15589447261437830 for Preoperative Corticosteroid Injection and Postoperative Infection Following De Quervain’s Release: A National Database Analysis by Mikayla P. Borusiewicz, Thompson Zhuang and David R. Steinberg in HAND
