# Supplementary material for: Preoperative Corticosteroid Injection and Postoperative Infection Following De Quervain’s Release: A National Database Analysis
Source: Hand (N Y). 2026 May 11:15589447261437830. Online ahead of print. doi: 10.1177/15589447261437830 (PMC13161037; doi:10.1177/15589447261437830)
Supplement: sj-docx-1-han-10.1177_15589447261437830 – Supplemental material for Preoperative Corticosteroid Injection and Postoperative Infection Following De Quervain’s Release: A National Database Analysis [file sj-docx-1-han-10.1177_15589447261437830.docx]

**Table S1.** Descriptions of codes used.

| **Description** | **ICD-9/10 or CPT codes** |
| --- | --- |
| *Explanatory Variable* | |
| Corticosteroid administration | CPT-J3300, CPT-J3301, CPT-J1020, CPT-J1030, CPT-J1040, CPT-J0702, CPT-J0704, CPT-J1100, CPT-J3302, CPT-J3303, CPT-J1094 |
| Injection into a tendon sheath | CPT-20550 |
| De Quervain’s tenosynovitis | ICD-9-D-72704, ICD-10-D-M654 |
| Trigger finger (*excluded*) | ICD-9-D-72703, ICD-10-D-M6530 through ICD-10-D-M65359 |
| *Included Surgeries* | |
| De Quervain’s release | CPT-25000 |
| *Comorbidities* | |
| Tobacco use | ICD-9-D-3051, ICD-9-D-V1582, ICD-10-D-F17220, ICD-10-D-F17221, ICD-10-D-F17223, ICD-10-D-F17228, ICD-10-D-F17229, ICD-10-D-F17290, ICD-10-D-F17291, ICD-10-D-F17293, ICD-10-D-F17298, ICD-10-D-F17299, ICD-10-D-Z720 |
| *Outcomes* | |
| Surgical site infection | ICD-9-D-68600, ICD-9-D-68609, ICD-9-D-6861, ICD-9-D-6868, ICD-9-D-9985, ICD-9-D-99851, ICD-9-D-99859, ICD-9-D-6823, ICD-9-D-6824, ICD-9-D-71103, ICD-9-D-71163, ICD-9-D-71164, ICD-9-D-73003, ICD-9-D-73004, ICD-9-D-73023, ICD-9-D-73024, ICD-9-D-73093, ICD-9-D-73094, ICD-10-D-L080, ICD-10-D-L0889, ICD-10-D-L980, ICD-10-D-L0889, ICD-10-D-T8140XA through ICD-10-D-T8144XS, ICD-10-D-K6811, ICD-10-D-L03111 through ICD-10-D-L03114, ICD-10-D-L03119, ICD-10-D-M00021 through ICD-10-D-M00049, ICD-10-D-M00121 through ICD-10-D-00149, ICD-10-D-M00221 through ICD-10-D-M00249, ICD-10-D-M00821 through ICD-10-D-M00849, ICD-10-D-M86131 through ICD-10-D-M86149, ICD-10-D-M86231 through ICD-10-D-M86249, ICD-10-D-M868X3 through ICD-10-D-M868X4 |
| Reoperation for surgical site infection | CPT-10060, CPT-10061, CPT-10180, CPT-11000, CPT-11001, CPT-20000, CPT-20005 |
| Wound dehiscence | ICD-9-D-99830, ICD-9-D-99831, ICD-9-D-99832, ICD-9-D-99833, ICD-10-D-T8130XA, ICD-10-D-T8130XD, ICD-10-D-T8130XS, ICD-10-D-T8131XA, ICD-10-D-T8131XD, ICD-10-DT8131XS, ICD-10-D-T8132XA, ICD-10-D-T8132XD, ICD-10-D-T8132XS, ICD-10-D-T8133XA, ICD-10-DT8133XD, ICD-10-D-T8133XS |

ICD-9/10 = International Classification of Diseases, Ninth or Tenth Revision. CPT = Current Procedural Terminology.

**Table S2.** Demographics before and after propensity score matching in sensitivity analysis.

| **Variable** | **Before Propensity Score Matching** | | | **After Propensity Score Matching** | | |
| --- | --- | --- | --- | --- | --- | --- |
|  | **Cohort** | | **p-value** | **Cohort** | | **p-value** |
|  | **Injection (n=4,164)** | **No Injection (n=118,305)** |  | **Injection**  **(n=4,164)** | **No Injection (n=4,164)** |  |
| Age group, n (%)  <45 years  45-54 years  55-64 years  ≥65 years | 1,165 (28.0)  958 (23.0)  1,125 (27.0)  916 (22.0) | 25,692 (21.7)  27,173 (23.0)  35,605 (30.1)  29,835 (25.2) | <0.001 | 1,165 (28.0)  958 (23.0)  1,125 (27.0)  916 (22.0) | 1,186 (28.5)  950 (22.8)  1,126 (27.0)  902 (21.7) | 0.95 |
| Sex, n (%)  Female  Male | 3,341 (80.2)  823 (19.8) | 97,681 (82.6)  20,624 (17.4) | <0.001 | 3,341 (80.2)  823 (19.8) | 3,353 (80.5)  811 (19.5) | 0.76 |
| Region, n (%)  Midwest  Northeast  South  West | 1,010 (24.3)  959 (23.0)  1,634 (39.2)  561 (13.5) | 31,810 (26.9)  25,570 (21.6)  46,634 (39.4)  14,291 (12.1) | <0.001 | 1,010 (24.3)  959 (23.0)  1,634 (39.2)  561 (13.5) | 998 (24.0)  959 (23.0)  1,655 (39.7)  552 (13.3) | 0.96 |
| Insurance plan, n (%)  Private  Medicare  Medicaid | 3,204 (76.9)  686 (16.5)  274 (6.6) | 92,804 (78.4)  17,876 (15.1)  7,625 (6.4) | 0.04 | 3,204 (76.9)  686 (16.5)  274 (6.6) | 3,275 (78.7)  651 (15.6)  238 (5.7) | 0.12 |
| Elixhauser comorbidity index, mean (SD) | 3.78 (3.33) | 3.85 (3.29) | 0.03 | 3.78 (3.33) | 3.58 (3.04) | 0.14 |

SD = standard deviation. Cohorts were propensity score matched for age, sex, region, insurance plan, Elixhauser comorbidity index, and history of tobacco use.

**Table S3.** Outcomes of sensitivity analysis for patients who did and did not receive injections within 60 days of surgical release.

| **Outcome** | **Cohort** | | **p-value*** |
| --- | --- | --- | --- |
|  | **Injection within 60 days (n=4,164)** | **No injection within 60 days (n=4,164)** |  |
| Surgical site infection, n (%) | 43 (1.0) | 37 (0.9) | 0.57 |
| Reoperation for infection, n (%) | 23 (0.6) | 15 (0.4) | 0.26 |
| Wound dehiscence, n (%) | 24 (0.6) | 11 (0.3) | 0.04 |
